# Ultrastructure, distribution, and transovarial transmission of symbiotic microorganisms in *Nysius ericae* and *Nithecus jacobaeae* (Heteroptera: Lygaeidae: Orsillinae)

**DOI:** 10.1007/s00709-012-0416-4

**Published:** 2012-05-16

**Authors:** Malgorzata Swiatoniowska, Antoni Ogorzalek, Aniela Golas, Anna Michalik, Teresa Szklarzewicz

**Affiliations:** 1 Department of Animal Developmental Biology, Zoological Institute, University of Wroclaw, Sienkiewicza 21, 50-335 Wroclaw, Poland; 2 Department of Developmental Biology and Morphology of Invertebrates, Institute of Zoology, Jagiellonian University, Gronostajowa 9, 30-387 Kraków, Poland; 3 Department of Genetics and Evolution, Institute of Zoology, Jagiellonian University, Gronostajowa 9, 30-387 Kraków, Poland

**Keywords:** Symbiotic microorganisms, Bacteriocytes, Transovarial transmission of symbionts, Heteroptera, Lygaeidae

## Abstract

The organization of the symbiotic system (i.e., distribution and ultrastructure of symbionts) and the mode of inheritance of symbionts in two species, *Nysius ericae* and *Nithecus jacobaeae* belonging to Heteroptera: Lygaeidae, are described. Like most hemipterans, *Nysius ericae* and *Nithecus jacobaeae* harbor obligate prokaryotic symbionts. The symbiotic bacteria are harbored in large, specialized cells termed bacteriocytes which are localized in the close vicinity of the ovaries as well as inside the ovaries. The ovaries are composed of seven ovarioles of the telotrophic type. Bacteriocytes occur in each ovariole in the basal part of tropharium termed the infection zone. The bacteriocytes form a ring surrounding the early previtellogenic oocytes. The cytoplasm of the bacteriocytes is tightly packed with large elongated bacteria. In the bacteriocytes of *Nysius ericae*, small, rod-shaped bacteria also occur. Both types of bacteria are transovarially transmitted from one generation to the next.

## Introduction

Symbiotic microorganisms commonly occur in the body of many insect species (for reviews, see, e.g., Buchner 1965; Douglas 1989 ; Moran and Telang 1998 ; Ishikawa 2003 ; Baumann 2005, 2006; Moran et al. 2008; Kikuchi 2009). As a rule, the presence of these symbionts in an insect’s body is connected with the unbalanced diet deficient in some essential nutrients, e.g., phloem sap consumed by most hemipterans is rich in carbohydrates but poor in amino acids, and the diet of blood-sucking insects is poor in B vitamins. The symbiotic microorganisms living in the bodies of the insects are able to synthesize and deliver to the hosts substances missing in the diet of the host. In most insects, prokaryotic symbionts occur, but in some aphids, scale insects (coccoids), leafhoppers, and planthoppers, eukaryotic “yeast-like symbionts” are present. The obligate symbionts may occur extracellularly in the lumen of midgut appendages (i.e., caeca, crypts) and intracellularly in the cells of midgut epithelium or in specialized cells termed bacteriocytes (= mycetocytes) (see, e.g., Buchner 1965; Kikuchi 2009 for further details). The latter are usually integrated into large organs termed bacteriomes (= mycetomes). As a rule, gut bacteria are transmitted to the next generation vertically (maternally) (Schneider 1940; Buchner 1965; Kikuchi 2009). They may be transmitted from the mother to the offspring in three ways: (1) through contamination of the egg surface by bacteria; (2) by feeding on parental excrements containing bacteria; and (3) by deposition of capsules which contain bacteria, onto the egg mass (see Rosenkranz 1939; Fukatsu and Hosokawa 2002; Kuechler et al. 2010, 2011 for further details). Recently, Kikuchi et al. (2005, 2007) have shown that gut symbionts of *Riptortus clavatus* and *Leptocorisa chinensis* (Heteroptera: Alydidae) are not vertically transmitted but each insect generation acquires the free-living bacteria from the environment. The symbionts that live in bacteriocytes (termed mycetomic symbionts) are transmitted to the next generation transovarially, i.e., via the cytoplasm of female germ cells (see Buchner 1965 for further details).

Buchner (1965) observed that in some hemipterans, more than one species of symbiotic microorganisms may occur. The symbionts that are always present in their hosts and are necessary for the host’s survival and reproduction are referred to as the primary symbionts (currently termed P-symbionts) by Buchner. For symbionts that only occur in some populations, Buchner called them accessory symbionts (currently termed facultative symbionts, secondary or S-symbionts). The P-symbionts occur intracellularly in bacteriocytes, whereas S-symbionts may occur both intracellularly (in bacteriocytes or in their epithelia) or extracellularly (e.g., free in the haemolymph or fat body) (see, e.g., Baumann 2005; Kikuchi 2009 for further details). The P-symbionts are always maternally (vertically) inherited by transovarial transmission, whereas the S-symbionts may be both transovarially and horizontally (i.e., between specimens of the same population) transmitted (Moran and Telang 1998; Fukatsu et al. 2000; Ishikawa 2003; Thao and Baumann 2004). It is believed that symbioses of insects and P-symbionts are the results of ancient infections (Baumann 2006). In contrast, the symbioses with S-symbionts are much younger than those with P-symbionts and are results of multiple infections (Thao and Baumann 2004).

Among hemipterans, the ultrastructure, distribution, and transmission of symbionts have only been extensively studied in Hemiptera: Sternorrhyncha (aphids, scale insects, psyllids, and whiteflies) (for reviews, see, Buchner 1965; Baumann 2005, 2006). There are relatively few papers concerning symbiotic associations between microorganisms and representatives of Hemiptera: Auchenorrhyncha (leafhoppers and planthoppers) and Heteroptera (for reviews, see, Buchner 1965; Kikuchi 2009). Histological and ultrastructural observations revealed that both in Hemiptera: Sternorrhyncha and Hemiptera: Auchenorrhyncha, symbionts are transovarially inherited. Older papers (Glasgow 1914; Kuskop 1924; Rosenkranz 1939; Schneider 1940; Buchner 1965) and some more recent papers (e.g., Kikuchi et al. 2005, 2007; Fukatsu and Hosokawa 2002; Kaiwa et al. 2010; Kuechler et al. 2010, 2011, 2012; Matsuura et al. 2012) concerning the symbiotic systems in heteropterans showed that they (i.e., distribution of symbionts, their transmission) are much more diverse than those in Hemiptera: Sternorrhyncha and Hemiptera: Auchenorrhyncha. In the majority of heteropterans, extracellular gut symbionts occur (see Kikuchi 2009 for further details). The mycetomic bacteria, however, have been found only in the family Cimicidae and in two families of the superfamily Lygaeoidea—Blissidae and Lygaeidae (see Schneider 1940; Buchner 1965; Kikuchi 2009; Kuechler et al. 2010, 2012 for further details). In the lygaeoid family Artheneidae (formerly treated as a subfamily of the Lygaeidae), the symbionts occur intracellularly in enlarged cells of the midgut epithelium that form “a mycetomic belt” (Kuechler et al. 2011). Moreover, results of recent papers (Kuechler et al. 2010, 2012; Matsuura et al. 2012) have revealed significant differences in the organization of symbiotic systems in the so far examined members of Lygaeidae belonging to subfamilies Orsillinae, Lygaeinae, and Ischnorhynchinae. The above fact indicates that in heteropterans, the distribution of symbiotic bacteria varies even among species of the same family. Thus, the first purpose of our study is to provide further information about the symbionts in the representatives of the Lygaeidae: Orsillinae (*Nysius ericae* and *Nithecus jacobaeae*). The second purpose of our study is to provide further information about symbiont transmission from the mother to the offspring.

## Material and methods

### Insects

Adult females of *Nysius ericae* (Schilling, 1829) from *Potentilla argentea* L. (Rosaceae), *Festuca pratensis* Huds. (Poaceae), *Rumex acetosella* L. (Polygonaceae), and *Oenothera biennis* L. (Onagraceae) were collected in Ruda Milicka (near Milicz, Poland), in the months of June and July from 2000 to 2004. Adult females of *Nithecus jacobaeae* (Schilling, 1829) were collected in Debki (near Puck, Poland) from *Senetio jacobaea* L. and *Hieracium pilosella* L. (Asteraceae), in the months of June and July from 2009 to 2011.

### Light and transmission electron microscopy

The ovaries of *Nysius ericae* were fixed in 4 % formaldehyde in phosphate buffered saline (PBS) for 30 min at room temperature and photographed under an Olympus BHS microscope equipped with phase contrast optics. For histological and ultrastructural observations, the dissected ovaries of *Nysius ericae* and the entire abdomens of *Nithecus jacobaeae* were fixed in 2.5 % glutaraldehyde in 0.1 M phosphate buffer (pH 7.4) for 24 h at room temperature, rinsed in the buffer with the addition of sucrose (5.8 g/100 ml), and postfixed in 1 % osmium tetroxide in the same buffer. After dehydration in a series of alcohol and acetone, the ovaries were embedded in Epon 812 epoxy resin (Serva, Heidelberg, Germany). Semithin sections (0.7 μm thick) were stained with 1 % methylene blue in 1 % borax and examined with Olympus BHS and Leica DMR light microscopes. Ultrathin sections (80 nm thick) were contrasted with uranyl acetate and lead citrate and examined in Zeiss EM 900 and Jeol JEM 100 SX transmission electron microscopes at 80 kV.

### Fluorescence microscopy

The ovaries of *Nysius ericae* were fixed in 4 % formaldehyde in PBS for 30 min, at room temperature. Next, they were rinsed in PBS, dehydrated, and embedded in Histocryl (Agar Scientific Ltd., Stansted, UK). Sections (0.7 μm thick) were stained with a 1:1 mixture of 4′,6-diamidino-2-phenylindoledihydrochloride (DAPI) (1 μg/ml; Sigma Chemical Co., St. Louis, USA) and propidium iodide (0.5 μg/ml; Serva) for 20 min in darkness, at room temperature. The sections were examined in an Olympus BHS epifluorescence microscope equipped with appropriate filters. After staining with DAPI, structures containing DNA (bacteria and cell nuclei) emit an intense white-blue fluorescence. After staining with propidium iodide, structures containing RNA (cytoplasm packed with ribosomes) emit an intense red fluorescence.

## Results

### Gross morphology of the ovaries of the adult female

**Fig. 1:**
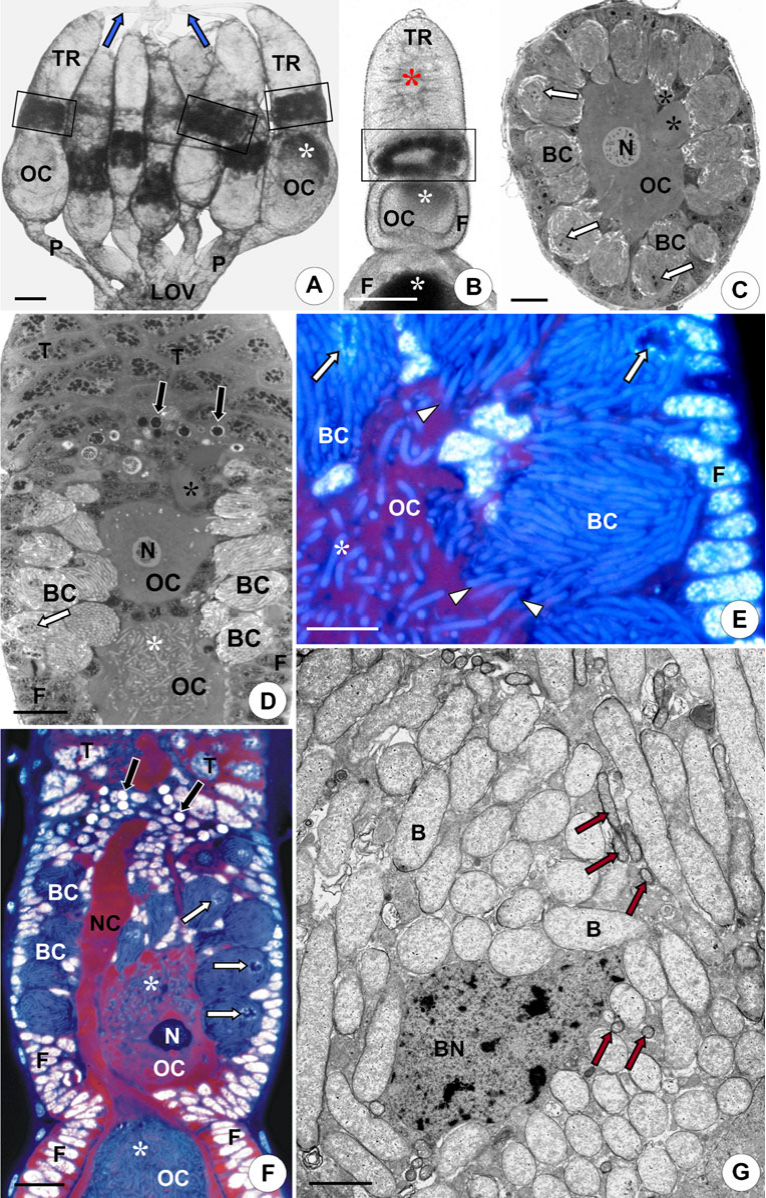
Organization of the infection zone in *Nysius ericae* and *Nithecus jacobaeae*. **a** *Nysius ericae*. Ovary of a young female. Terminal filaments (*blue arrows*), infection zone (*in frames*), accumulation of symbiotic microorganisms (*white asterisk*), lateral oviduct (*LOV*), oocyte (*OC*), pedicel (*P*), tropharium (*TR*). Phase contrast, *bar* = 100 μm. **b** *Nysius ericae*. Fragment of the ovariole. Note the infection zone (*in frame*). Accumulations of symbiotic bacteria in the oocyte cytoplasm (*white asterisks*), trophic core (*red asterisk*), follicular epithelium (*F*), oocyte (*OC*), tropharium (*TR*). Phase contrast, *bar* = 100 μm. **c** *Nithecus jacobaeae.* Cross section through the early previtellogenic oocyte (*OC*) that is surrounded by bacteriocytes (*BC*). Nuclei of bacteriocytes (*white arrows*), nutritive cords (*black asterisks*), oocyte nucleus (*N*). Methylene blue, *bar* = 20 μm. **d** *Nithecus jacobaeae.* Fragment of the ovariole containing the infection zone. Early meiotic oocytes (*black arrows*), nucleus of bacteriocyte (*white arrow*), nutritive cord (*black asterisk*), accumulation of symbiotic bacteria in the oocyte cytoplasm (*white asterisk*), bacteriocytes (*BC*), follicular epithelium (*F*), oocyte nucleus (*N*), early previtellogenic oocytes (*OC*), syncytial lobes containing several trophocyte nuclei (*T*). Methylene blue, *bar* = 20 μm. **e**, **f** *Nysius ericae*. Fragment of the ovariole containing the infection zone (longitudinal section). Accumulations of symbiotic bacteria in the oocyte cytoplasm (*white asterisks*), early meiotic oocytes (*black arrows*), nuclei of bacteriocytes (*white arrows*), bacteria that enter the oocyte cytoplasm (*white arrowheads*), bacteriocyte (*BC*), follicular epithelium (*F*), oocyte nucleus (*N*), nutritive cord (*NC*), oocyte (*OC*), syncytial lobes containing several trophocyte nuclei (*T*). DAPI + propidium iodide, **e** *bar* = 10 μm; **f** *bar* = 20 μm. **g** *Nysius ericae*. Fragment of the ovariolar bacteriocyte. Small, rod-shaped bacteria (*red arrows*), large, elongated bacteria (*B*), bacteriocyte nucleus (*BN*). TEM, *bar* = 2 μm

**Fig. 2:**
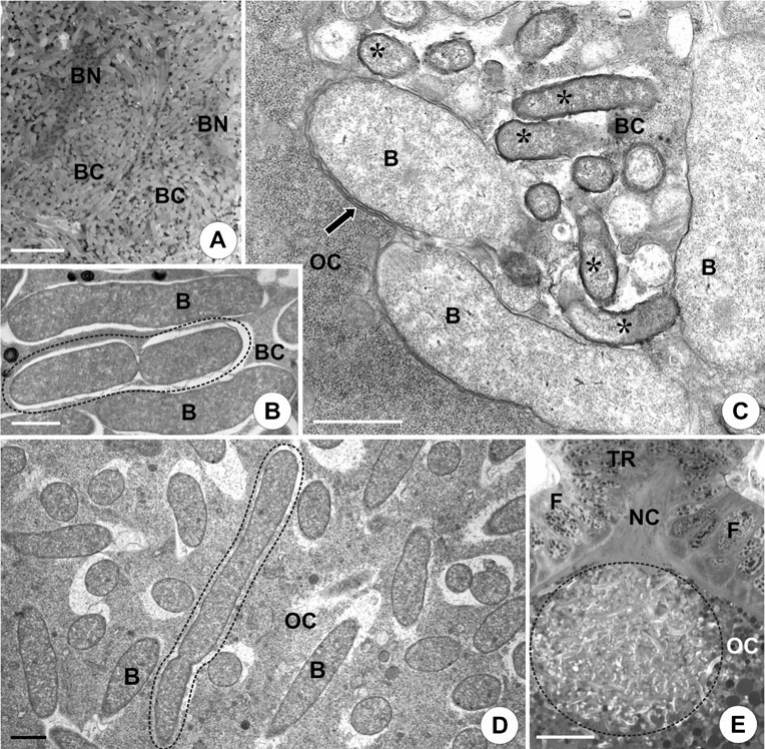
**a** *Nithecus jacobaeae.* Fragment of the bacteriome. Bacteriocyte (*BC*), bacteriocyte nucleus (*BN*). Methylene blue, *bar* = 20 μm. **b** *Nithecus jacobaeae.* Fragment of the bacteriocyte (*BC*). Note dividing large, elongated bacteria (*encircled with dotted line*). Large, elongated bacteria (*B*). TEM, *bar* = 2 μm. **c** *Nysius ericae*. Fragment of the infection zone. Both types of bacteria leave the bacteriocyte (*BC*) and enter the oocyte (*OC*). *Black arrow* indicates the perisymbiotic membrane. Large, elongated bacteria (*B*), small, rod-shaped bacteria (*black asterisks*). TEM, *bar* = 1 μm. **d** *Nysius ericae*. Large, elongated bacteria (*B*) dispersed in the cytoplasm of previtellogenic oocyte (*OC*). Note dividing bacteria (*encircled with dotted line*). TEM, *bar* = 2 μm. **e** *Nithecus jacobaeae.* Fragment of the ovariole (longitudinal section). Note “the symbiotic ball” (*encircled with dotted line*) located at the anterior pole of the vitellogenic oocyte (*OC*), follicular epithelium (*F*), nutritive cord (*NC*), tropharium (*TR*). Methylene blue, *bar* = 20 μm

The ovaries of *Nysius ericae* and *Nithecus jacobaeae* are located ventro-laterally in the abdomen. Each of them is composed of seven ovarioles of the telotrophic type (Fig. 1a). Like ovarioles of the remaining heteropterans, individual ovarioles of *Nysius ericae* and *Nithecus jacobaeae* are composed, from apex to base, of four well-defined regions: a terminal filament, trophic chamber (tropharium), vitellarium, and ovariolar stalk (pedicel) (Fig. 1a). The terminal filaments join together forming a ligament (Fig. 1a). The latter attaches the ovary to the lobe of the fat body. The ovariolar stalk connects the ovariole to the lateral oviduct (Fig. 1a). The tropharium is composed of numerous syncytial lobes containing several trophocyte nuclei embedded in the common cytoplasm (Fig. 1d). The syncytial lobes are radially arranged around the trophic core (Fig. 1b). Apart from these elements, in the basal region of the trophic chamber, there are early meiotic oocytes, early previtellogenic oocytes, and somatic prefollicular cells (Fig. 1d, f). Since the early previtellogenic oocytes are surrounded by large cells termed bacteriocytes (Fig. 1c–f), this part of the ovariole is termed “an infection zone” (Fig. 1a, b) (see below). The vitellarium of the mature female is composed of one or two oocytes (Fig. 1a, b) that are surrounded by a single layer of follicular cells (Figs. 1d–f and 2e). The oocytes in the vitellarium undergo three subsequent stages: previtellogenesis (i.e., synthesis and accumulation of RNAs), vitellogenesis (synthesis and accumulation of reserve substances), and choriogenesis (synthesis and secretion of precursors of egg envelopes). The oocytes (early meiotic oocytes, early previtellogenic oocytes, previtellogenic oocytes, and vitellogenic oocytes) are connected with the trophic core by means of nutritive cords (Figs. 1d, f and 2e) (for a detailed description of ovaries of heteropterans, see Simiczyjew et al. 1998).

### Ultrastructure, distribution and transovarial transmission of symbiotic microorganisms

The ovaries of *Nysius ericae* and *Nithecus jacobaeae* are accompanied by large, paired, irregularly shaped organs, called bacteriomes. Since only dissected ovaries of *Nysius ericae* have been examined, the structure of their bacteriomes has not been investigated. Bacteriomes of *Nithecus jacobaeae* are composed of several huge bacteriocytes (Fig. 2a). The cytoplasm of these cells is tightly packed with large, elongated bacteria (Fig. 2a, b). These microorganisms measure about 10 μm in length and about 1.5 μm in diameter.

In both examined species, apart from bacteriomes, symbiotic bacteria also occur in ovariolar bacteriocytes within the infection zone (Figs. 1a–g and 2c). The latter surround the early previtellogenic oocytes forming a ring around the ovariole (Fig. 1b, c). The cytoplasm of ovariolar bacteriocytes is tightly packed with large, elongated bacteria (Fig. 1g) that are highly fluorescent after DAPI staining (Fig. 1e, f). In ovariolar bacteriocytes of *Nysius ericae* apart from large, elongated bacteria, a few small, rod-shaped bacteria are present (Figs. 1g and 2c). The smaller bacteria are approximately 1 μm in length and about 0.3 μm in diameter. When the bacteria start to invade the oocytes, the plasma membranes of bacteriocytes and oocytes closely adhere to each other (Figs. 1e and 2c). Both larger (Figs. 1e and 2c) and smaller microorganisms (Fig. 2c) are gradually released from the bacteriocyte cytoplasm. The bacteria protrude from the bacteriocyte and penetrate the oocyte cytoplasm (Figs. 1e and 2c). Entering the oocyte, the bacteria are surrounded by the plasma membrane of the oocyte (Fig. 2c). As a consequence, the microorganisms are surrounded by an additional, host-derived membrane termed the perisymbiotic membrane. In the previtellogenic oocytes, the bacteria are dispersed in the entire ooplasm (Figs. 1b, d–f and 2d). In vitellogenic oocytes, they accumulate at their anterior poles and finally form a characteristic “symbiont ball” (Fig. 2e). In the ooplasm (Fig. 2d), like in the bacteriocytes (Fig. 2b), the bacteria multiply by binary fission.

## Discussion

Histological studies by Buchner (1965, 1966, 1967) as well as more recent ultrastructural studies (Cheng and Hou 2001; Szklarzewicz and Moskal 2001; Szklarzewicz et al. 2006, 2010, 2012; Sacchi et al. 2008; Kuechler et al. 2010, 2011, 2012; Matsuura et al. 2012 ) have shown that hemipterans possessing mycetomic symbionts developed variable modes of their transmission to the next generations. Moreover, it appeared that in closely related groups (e.g., in closely related families of scale insects such as Monophlebidae and Marchalinidae), inheritance of symbionts may take a different course (Szklarzewicz et al. 2006, 2012). First, symbionts may infest young germ cells, i.e., cystocytes before differentiation into oocytes and trophocytes (in some scale insects) or older oocytes, i.e., during the vitellogenic or choriogenic stage (in most hemipterans). Second, the ovaries may be infested by whole intact bacteriocytes (in whiteflies) or by microorganisms released from bacteriocyte cytoplasm (in most hemipterans). Third, the symbionts may enter the cytoplasm of follicular cells surrounding the oocyte (in some scale insects and planthoppers) or the symbionts may migrate between neighboring follicular cells (in most hemipterans). Fourth, the bacteria enter the oocyte cytoplasm immediately after the passage through the follicular epithelium (in aphids) or the bacteria gather in the deep depression of the oolemma and stay out of the oocyte till the end of its growth (in most hemipterans). Ultrastructural studies showed that in *Nysius ericae* and *Nithecus jacobaeae*, young oocytes (i.e., early previtellogenic) located in the region of the infection zone are infested by symbiotic microorganisms. Thus, the mode of the oocyte infection in these species markedly differs from the modes described in other hemipterans. It should be noted that other European and Japanese *Nysius* species have a similar organization of the infection zone as the organization found in *Nysius ericae* and *Nithecus jacobaeae* (Schneider 1940; Matsuura et al. 2012). A similar organization is also found in several other representatives of the superfamily Lygaeoidea, namely in *Kleidocerys resedae* (Lygaeidae: Ischnorhynchinae) (Kuechler et al. 2010), *Arocatus longiceps* (Lygaeidae: Lygaeinae) (Kuechler et al. 2012), *Orsillus depressus* and *Ortholomus punctipennis* (Lygaeidae: Orsillinae) (Kuechler et al. 2012), *Chilacis typhae* (Artheneidae) (Kuechler et al. 2011), and *Ischnodemus sabuleti* (Blissidae) (Kuechler et al. 2012). In contrast to ovariolar bacteriocytes, the organization and localization of bacteriomes in the above species markedly differ (Kuechler et al. 2010, 2011, 2012; Matsuura et al. 2012). In representatives of the Lygaeidae: Orsillinae (this study; Schneider 1940; Matsuura et al. 2012; Kuechler et al. 2012), Lygaeidae: Lygaeinae (Kuechler et al. 2012), and Blissidae (Kuechler et al. 2012), the bacteriomes are paired. They may be compact (i.e., tubular) or composed of two or three parts (Kuechler et al. 2012). The bacteriomes may be localized in the vicinity of the ovaries or close to the body wall (Kuechler et al. 2012). In *K. resedae* (Lygaeidae: Ischnorhynchinae), the bacteriome is unpaired and situated near the midgut (Kuechler et al. 2010). According to Matsuura et al. (2012) and Kuechler et al. (2012), the differences in the localization and morphology of bacteriomes in lygaeoid bugs suggest that the bacteriomes evolved independently from each other. The symbiotic system in *C. typhae* (Artheneidae) is of special interest because (1) the typical bacteriocytes do not occur in this species and (2) the symbiotic bacteria are housed in the cytoplasm of enlarged cells of the midgut epithelium. Although the bacteria are not harbored in bacteriocytes, they are, like mycetomic symbionts, transovarially transmitted to the next generation. Based on this observation, Kuechler et al. (2011) considered the symbiotic system in *C. typhae* as an evolutionarily intermediate state between the gut symbiosis and bacteriocyte symbiosis. It should be stressed that this hypothesis is substantiated by the presence of similar phenomenon in scale insects (Hemiptera: Sternorrhyncha). The symbionts in scale insects, like in other sternorrhynchans, are harbored in bacteriocytes and are transovarially transmitted to the offspring (see, e.g., Buchner 1965; Fukatsu and Nikoh 2000; von Dohlen et al. 2001; Szklarzewicz et al. 2006, 2010, 2012; Matsuura et al. 2009). The one exception to this rule has been observed by Buchner (1967) in *Marchalina hellenica* (Marchalinidae). In this species, the bacteria do not occur in bacteriocytes but in modified cells of the midgut epithelium. Like in the remaining scale insects, the bacteria are transovarially transmitted to the next generation (Buchner 1967; Szklarzewicz et al. 2012). According to Buchner (1967), symbionts are transported from the midgut epithelium to the ovaries by means of “temporary” bacteriocytes. Thus, in the light of the above facts, the symbiotic system in *M. hellenica* may also be regarded as an intermediate condition between gut and bacteriocyte symbiosis.

Recent studies on symbiotic systems of lygaeoid bugs revealed that apart from differently organized bacteriomes, these insects are characterized by diverse symbionts. Thus far, within the Heteroptera: Lygaeoidea, the symbionts have been molecularly analyzed in the representatives of three families, Artheneidae (Kuechler 2011), Lygaeidae (subfamilies Lygaeinae, Orsillinae, and Ischnorhynchinae) (Kuechler et al. 2010, 2012; Matsuura et al. 2012), and Blissidae (Kuechler et al. 2012). Matsuura et al. (2012) examined symbiotic bacteria in four species of the genus *Nysius* (Lygaeidae: Orsillinae) and revealed that their symbionts represent γ-proteobacteria. Kuechler et al. (2010, 2011, 2012) indicated that symbionts of *C. typhae* (Artheneidae), *K. resedae* (Lygaeidae: Ischnorhynchinae), *I. sabuleti* (Blissidae), *A. longiceps* (Lygaeidae: Lygaeinae), *Belonochilus numenius*, *O. depressus*, *O. punctipennis* (all three Lygaeidae: Orsillinae) also belong to γ-proteobacteria. However, phylogenetic analysis inferred from the 16S rRNA and the *groEL* gene sequences indicated that symbionts in the examined Lygaeoidea are not closely related to each other (Kuechler et al. 2010, 2011, 2012; Matsuura et al. 2012). Furthermore, phylogenetically distinct symbionts may occur in members of the same subfamily Orsillinae (Kuechler et al. 2012). Kuechler et al. (2010, 2011, 2012) and Matsuura et al. (2012) suggested that symbionts of lygaeoids have been acquired independently from each other. Moreover, on the basis of the similarity in the 16S rRNA and *groEL* gene sequences of symbionts of *C. typhae* and free-living bacteria, Kuechler et al. (2011) hypothesized that symbiotic association between the *Chilacis* and its symbionts is a result of a more recent infection with a free-living ancestor. This in turn corresponds well with the hypothesis concerning “the intermediate state” of the symbiotic system in *Chilacis* (see above).

Ultrastructural observations showed that in *Nysius ericae* apart from large, elongated bacteria, small, rod-shaped bacteria are present. The rod-shaped bacteria occur in the cytoplasm of the same bacteriocytes as larger bacteria and infect oocytes in the same way and at the same time as the larger ones. The function of these bacteria remains unknown. They may play a positive role for their host. It also cannot be excluded that they may be pathogens. Since we did not observe detectable aberrations during the development of *Nysius ericae*, the latter possibility seems to be rather unlikely. The localization of these bacteria in bacteriocytes as well as their transovarial transmission seems to indicate that small, rod-shaped bacteria represent facultative symbionts (S-symbionts) of *Nysius ericae*. Both the size and ultrastructure of the smaller rod-shaped bacteria in *Nysius ericae* suggest that these bacteria may belong to the widely widespread within arthropods genus *Wolbachia*. This assumption is in agreement with the observation of Kikuchi and Fukatsu (2003) that numerous heteropteran species are infected by *Wolbachia*. Among 134 examined species from 19 families of heteropterans, these authors detected *Wolbachia* infections in 47 species from 13 families. The *Wolbachia* bacterium has also been found in the Japanese member of the genus *Nysius*, namely *Nysius plebejus*. The biological function of *Wolbachia* in *Nysius plebejus* is not clear because, as suggested by Kaiwa et al. (2010), some of *Wolbachia* infections in heteropterans may be of parasitic or commensalistic nature. It should be noted that Kuechler et al. (2010) identified in the body (e.g., in the fat body, bacteriocytes, and oocytes) of *K. resedae* (Lygaeidae: Ischnorhynchinae) the bacterium *Rickettsia* as well as the bacterium *Wolbachia.* However, neither *Wolbachia* nor *Rickettsia* had a negative influence on reproduction of the host insect. Thus, to clarify the microbiological nature and function of small, rod-shaped bacteria, further studies on other *Nysius* species as well as on specimens of *Nysius ericae* from other populations are required.
